# Cytotoxicity of Seaweed Compounds, Alone or Combined to Reference Drugs, against Breast Cell Lines Cultured in 2D and 3D

**DOI:** 10.3390/toxics9020024

**Published:** 2021-01-31

**Authors:** Fernanda Malhão, Alice Abreu Ramos, Ana Catarina Macedo, Eduardo Rocha

**Affiliations:** 1 Institute of Biomedical Sciences Abel Salazar (ICBAS), University of Porto (U.Porto), Rua de Jorge Viterbo Ferreira 228, 4050-313 Porto, Portugal; fcmalhao@icbas.up.pt (F.M.); ramosalic@gmail.com (A.A.R.); acfpmacedo@gmail.com (A.C.M.); 2 Interdisciplinary Center for Marine and Environmental Research (CIIMAR), University of Porto (U.Porto), Avenida General Norton de Matos, 4450-208 Matosinhos, Portugal

**Keywords:** breast cancer, combinatory therapy, drug screening, in vitro, multicellular aggregates

## Abstract

Seaweed bioactive compounds have shown anticancer activities in in vitro and in vivo studies. However, tests remain limited, with conflicting results, and effects in combination with anticancer drugs are even scarcer. Here, the cytotoxic effects of five seaweed compounds (astaxanthin, fucoidan, fucosterol, laminarin, and phloroglucinol) were tested alone and in combination with anticancer drugs (cisplatin—Cis; and doxorubicin—Dox), in breast cell lines (three breast cancer (BC) subtypes and one non-tumoral). The combinations revealed situations where seaweed compounds presented potentiation or inhibition of the drugs’ cytotoxicity, without a specific pattern, varying according to the cell line, concentration used for the combination, and drug. Fucosterol was the most promising compound, since: (i) it alone had the highest cytotoxicity at low concentrations against the BC lines without affecting the non-tumoral line; and (ii) in combination (at non-cytotoxic concentration), it potentiated Dox cytotoxicity in the triple-negative BC cell line. Using a comparative approach, monolayer versus 3D cultures, further investigation assessed effects on cell viability and proliferation, morphology, and immunocytochemistry targets. The cytotoxic and antiproliferative effects in monolayer were not observed in 3D, corroborating that cells in 3D culture are more resistant to treatments, and reinforcing the use of more complex models for drug screening and a multi-approach that should include histological and ICC analysis.

## 1. Introduction

Breast cancer (BC) is the most diagnosed cancer among women in high human development index countries and a leading cause of cancer death among females [1,2]. BC treatment involves different therapeutic approaches based mainly on the extent of the disease and the tumor characteristics [3]. It is a very heterogeneous cancer type, presenting different molecular subtypes which are associated with different prognostics. The determination of the molecular subtype is commonly performed by immunohistochemistry and/or genetic analyses and its classification is related to the positivity or negativity for estrogen and progesterone receptors (ER and PR), as well as for eventual (over)expression of the oncogene human epidermal growth factor receptor 2 (HER-2). The main molecular subtypes are: (a) luminal (ER and PR positive); (b) HER-2 enriched (ER, PR negative, and HER-2 overexpression), and (c) triple-negative breast cancer (TNBC) (ER, PR, and HER-2 negative) [4,5,6]. For luminal and HER-2 subtypes, there are effective therapeutic drugs [7], such as the well-established ER antagonist tamoxifen for hormone-positive tumors [8] and the antibody trastuzumab, to HER2 subtype [9]. Patients with TNBC are generally considered as high-risk patients, presenting the poorest prognosis as they cannot benefit from target therapies and therefore, the recommended treatment approach for this type of patients is usually systemic chemotherapy [3].

Transversal to all cancer types is the problematic of drug resistance (innate or acquired) [10] and the high cumulative drug toxicity of some chemotherapeutics on non-cancer cells [11]. Therefore, there has been a great struggle for finding new drugs or drug adjuvants to overcome both drug resistance and toxicity. This topic is a hotspot in the pharmaceutical industry and in the scientific community. In this vein, one therapeutical approach that has been applied is the use of multi-drug combinations that target non-overlapping signaling pathways [12], with the intention to improve the coverage of therapeutic responses and reduce the prospect of resistance [13] and toxicity [14]. This approach has been applied in many cancer types including BC, especially in TNBC or in metastatic BC [15]. The combination therapy revealed efficacy in lowering drugs’ doses or acting in a synergistic way, potentiating drugs’ effects, or even reducing toxicity against normal cells [16,17,18].

Although it is almost unknown by the community in general, 50–60% of the drugs approved for cancer treatment are natural compounds and their derivatives [19,20]. The marine environment is an immense reservoir of natural compounds with a huge chemical and biological diversity. Among the rich marine flora, there has been a growing interest in the pharmacological activities of marine macroalgae (seaweeds), especially in their bioactive metabolites that can modulate the mechanisms involved in cancer. Anticancer activities of these compounds have been associated with inhibition of cell proliferation, proapoptotic, antiangiogenic, and anti-metastasis effects [21,22,23,24,25]. Interestingly, seaweeds have been used for centuries in Traditional Chinese and Japanese Folk Medicines in attempts to treat BC [26,27]. Data from several epidemiological and experimental studies confirmed the potential effects of seaweed dietary consumption in BC prevention [28,29,30]. Various studies reported the use of natural products in combination therapy with anticancer drugs [31,32,33,34,35]. When referring to seaweed compounds, the knowledge of interactions with drugs is limited to a few in vitro [36,37,38] and in vivo studies [39,40]. Furthermore, when considering the exploitation of the antioxidant properties of seaweed compounds, it should be remembered that the intake of antioxidants during chemotherapy is controversial, specifically in relation to BC. Evidences suggest that the effects, beneficial or not, are related to the dose intake and type of antioxidant [41]. While some authors advised against the intake of antioxidants during BC treatment [42], others showed that the administration of antioxidants in the first six months after BC diagnosis could reduce the risk of mortality and tumor recurrence [43].

Screening for new anticancer drugs is often performed using in vitro studies, and typically with cancer cell lines cultivated in monolayer [44]. Nowadays, there is a consensus in the literature that the use of more complex in vitro models, such as three-dimensional (3D) cell cultures, better simulates the in vivo tumor microenvironment [45,46]. The arrangement of cells into 3D cell multicellular aggregates (MCAs) is associated with a more functional state and promotes different gradients of nutrients and oxygen supply [47,48]. Additionally, cells cultured in 3D are supposed to be more resistant to drug treatments [49,50,51].

When referring to the screening of effects of seaweed bioactive compounds in BC cell lines, there are no systematic studies using a panel of cancer cell lines with distinct biological characteristics while comparatively testing normal breast cell lines. Also, in what concerns combinations with drugs, it is poorly explored if the cell line characteristics can influence the type of response.

In concord with the current state of the art, it is worth exploring the anticancer properties of selected seaweed compounds alone and in combination with reference drugs in a panel of breast cancer cell lines. For that, we selected three BC cell lines representative of the main BC subtypes: (i) MCF7 (ER+, PR+, HER-2–), corresponding to the most common BC type—Luminal A; (ii) SKBR3 (ER–, PR–, HER-2+), representing the HER-2 subtype; and (iii) MDA-MB-231, a triple-negative cell line (ER–, PR–, HER-2–), equivalent to TNBC [52,53]. We also included a non-tumor breast cell line (iv) MCF12A [52].

For this combinatory panel screening, we selected five brown seaweed bioactive compounds belonging to different chemical groups: (i) carotenoids: astaxanthin (Asta); (ii) polysaccharides: fucoidan (Fc) and laminarin (Lm); (iii) sterols: fucosterol (Fct); and (iv) phlorotannins: phloroglucinol (Phg).

### 1.1. Carotenoids—Astaxanthin (Asta)

Carotenoids are fat-soluble organic pigments, naturally occurring in phototrophic organisms [54]. Asta is a xanthophyll carotenoid without vitamin A [55] present in diverse marine organisms, including brown seaweeds [56]. Compared with other carotenoids, its chemical structure possesses a special feature: two keto groups on each ring structure, which enhances its antioxidant properties. That is why it is called the “super antioxidant” [57]. Some anticancer activities of Asta have been reported, such as inhibition of cell proliferation [58,59] and apoptosis induction [58,60,61]. In BC cell lines, Asta significantly reduced proliferation rates and inhibited cell migration compared to control normal breast epithelial cells [62]. Asta was described as having a potent effect in inhibiting tumor growth due to its anti-inflammatory properties [63].

### 1.2. Polysaccharides—Fucoidan (Fc) and Laminarin (Lm)

Sulphated polysaccharides are a major constituent of seaweeds’ cell walls that have attracted much attention as functional additives in the pharmaceutical, food and cosmetic industries [64]. Fc is a complex sulphated polysaccharide, with many biological activities: antioxidant, anticoagulant, antiviral, immunomodulatory, antiproliferative, antilipidemic, anti-inflammatory, and anti-metastasis [22,65,66,67]. Accumulating data show the anticancer effects of Fc in several cancer cell lines [68,69,70]. In BC cell lines, Fc induced the apoptosis pathway in MCF7 [71,72,73] and MDA-MB-231 cells [73,74], inhibiting cell growth in both cell lines [73]. Also, colony formation was inhibited by this compound in the BC cell line T47D [73,75]. Fc was also pointed out as having a regulatory role in migration and invasion in MDA-MB-231 [76]. In vitro co-exposure using Fc with cisplatin, tamoxifen, or paclitaxel, potentiated the effect of the drug in MCF7 and MDA-MB-231 [73]. Moreover, case studies have shown that the use of Fc as alternative medicine in mouse models and human clinical trials seems to alleviate the side effects of anticancer chemotherapy [70].

Lm is a water-soluble polysaccharide, corresponding to a storage glucan. Glucans are Food and Drug Administration approved compounds for lowering cholesterol levels [77] and they have been described to promote anticancer immunity [78]. Evidence have shown that Lm has anticancer activity in HT 20 human colon cells by inducing apoptosis in a dose-dependent way [77,79], and also lead to apoptosis through mitochondrial pathway in human colon cancer cell line LOVO [80]. Laminarins and their sulphated derivatives inhibited proliferation [81], colony formation, and migration in several human cell lines including BC ones [82].

### 1.3. Sterols—Fucosterol (Fct)

Phytosterols represent a class of cholesterol-like molecules that integrate the cellular membranes of plants and algae, having a role in the regulation of membrane permeability [83]. Fct has been mentioned as anti-inflammatory, antibacterial, antifungal, antidiabetic, antidepressant, anticancer, antioxidant, and protective against a wide range of diseases [84,85]. Fct had a cytotoxic effect in T47D breast cell line [86], induced mitochondrial-mediated apoptosis, migration, inhibition, and downregulation of m-TOR/PI3K/Akt signalling pathway in MCF7 [87]. Fct containing fractions presented cytotoxicity against human colon and BC cell line (T47D), without inducing cytotoxic effects on the normal cell line [86], and also reduced cell proliferation and induced apoptosis in MCF7 and MDA-MB-231 cell lines but these effects were not so evident in the non-tumoral cell line CHO [88].

### 1.4. Phlorotannins–Phloroglucinol (Phg)

Phg is a polyphenolic compound whose chemical structure includes an aromatic phenyl ring with three hydroxyl groups. Its biological activities include antioxidant and anti-inflammatory actions [89,90]. The former seems to be related to free radical-scavenging and metal chelation properties. Phg induced cytotoxicity through caspases activation in MDA-MB-231 BC cell line [91] and suppressed metastasis in invasion assays with the same cell line [92]. Additionally, in assays with BC cell lines, Phg suppressed sphere formation, anchorage-independent colony formation and in vivo tumorigenicity, and decreased the cancer stem cell population [92].

As reference anticancer drugs, we chose two drugs used for treating many cancer types, including BC [93,94]: (i) cisplatin (Cis), an alkylating agent that damage the structure of DNA through the crosslinking forming platinum-DNA adducts that interfere with DNA transcription and replication, resulting in cell death; and (ii) doxorubicin (Dox), an anthracycline antibiotic with no completely clear mechanisms of action, but it has been reported to cause oxidative stress and block RNA transcription by intercalation into DNA bases [95]. Both are highly effective drugs, but with associated side effects and drug resistance [96,97,98]. Cis can cause nephrotoxicity, neurotoxicity and hearing impairments [96]. Dox is vastly used in BC adjuvant and neoadjuvant chemotherapy [99], but it also elicits cardiotoxicity, secondary leukaemia, myelosuppression, intestinal epithelium lesions, and chemotherapy-related infertility [100].

In view of the above, this study aimed to evaluate the cytotoxic activity of the seaweed bioactive compounds Asta, Fc, Fct, Lm, and Phg, alone or combined with Cis and Dox, in three BC cell lines and one non-tumorous breast line, in monolayer culture (2D). The most promising combination of seaweed compound plus drug in monolayer was chosen to be investigated as to viability and proliferation, using a comparative approach with two in vitro systems (2D-monolayers versus 3D–MCAs).

## 2. Materials and Methods

### 2.1. Cell Lines and Baseline Culture Conditions

MCF7 was acquired from the European Collection of Authenticated Cell Cultures (ECACC). SKBR3 cell line was kindly provided by Professor Carmen Jerónimo (Portuguese Oncology Institute–Porto, Portugal). MCF12A and MDA-MB-231 cell lines were purchased from the American Tissue Culture Collection (ATCC). MCF7, MDA-MB-231, and SKBR3 were cultivated in high glucose Dulbecco’s modified Eagle’s medium (DMEM), without glutamine and phenol red, supplemented with 10% Fetal Bovine Serum (FBS) and 1% antibiotics solution penicillin/streptomycin (pen/strep) (10,000 U/mL/10,000 μg/mL, respectively). MCF12A was cultivated in DMEM/Ham’s Nutrient Mixture F12 (DMEM/F12) medium without phenol red and supplemented with 20 ng/mL human epidermal growth factor (hEGF), 100 ng/mL cholera toxin, 0.01 mg/mL insulin, 500 ng/mL hydrocortisone, 10% FBS, and 1% of the same antibiotic solution. All cell lines were cultivated in T75 cm^3^ culture flasks (Orange Scientific, Braine-l’Alleud, Belgium) and maintained in the incubation chamber MCO 19AIC (Sanyo, Tokyo, Japan), with 5% CO_2_, at 37 °C. For cells’ growth and maintenance, the culture medium was replaced every two days. Cells were regularly observed using an inverted phase-contrast microscope CKX41 (Olympus, Tokyo, Japan). The experiments were performed with cells under passage number 30. At 80% of cell confluence, monolayer cells were subcultured using trypsin/ethylenediamine tetraacetic acid (EDTA) (trypsin/EDTA) solution (0.25/0.02 in %).

### 2.2. Chemicals and Solutions

Dimethyl sulfoxide (DMSO) was purchased from VWR Chemicals (Solon, OH, USA). 3-(4,5-Dimethyl-2-thiazolyl)-2,5-diphenyl-2H-tetrazolium bromide (MTT), astaxanthin (SML0982, CAS Number: 472-61-7, MW: 596.84), fucoidan (F8190, CAS Number: 9072-19-9, MW: not determined by the supplier); fucosterol (F5379, CAS Number: 17605-67-3, MW: 412.69), laminarin (L9634, CAS Number: 9008-22-4, MW: not determined by the supplier); phloroglucinol (79,330, CAS Number: 108-73-6, MW:126.11), cisplatin (C2210000, CAS Number: 15663-27-1; MW:300.05), doxorubicin (D1515, CAS Number: 25316-40-9, MW: 579.98); insulin (I2643); cholera toxin (C8052), hydrocortisone (H088), and hEGF (E9644) were obtained from Sigma Aldrich (St.Louis, MO, USA). DMEM (F045), FBS, pen/strep, trypsin/EDTA, were acquired from Biochrom KG (Berlin, Germany). DMEM/F12 was obtained from GE Healthcare (Chicago, IL, USA). Resazurin (14,322) was purchased from Cayman (AnnArbor, MI, USA). Cell Proliferation ELISA and BrdU (5′-bromo-2′-deoxyuridine) kit (colorimetric) were acquired from Roche (Basel, Switzerland). All other reagents and chemicals used were analytical grade.

Stock solutions of astaxanthin (Asta), fucosterol (Fct), phloroglucinol (Pgh), and doxorubicin (Dox) were prepared in DMSO, while those of fucoidan (Fc) and laminarin (Lm) were prepared in supplemented cell culture medium. All those stock solutions were kept at −20 °C until used, except for the stock solutions of Fc and Lm that were prepared immediately before use. The stock solution of cisplatin (Cis) was prepared in a 0.9% NaCl solution and kept at 4 °C up to 1 month. Exposure solutions were prepared immediately before each experiment by diluting the stock solutions into the appropriate volume of respective fresh culture medium ensuring a final concentration of 0.1% DMSO.

### 2.3. Cell Exposures

#### 2.3.1. Study Design

**Figure 1:**
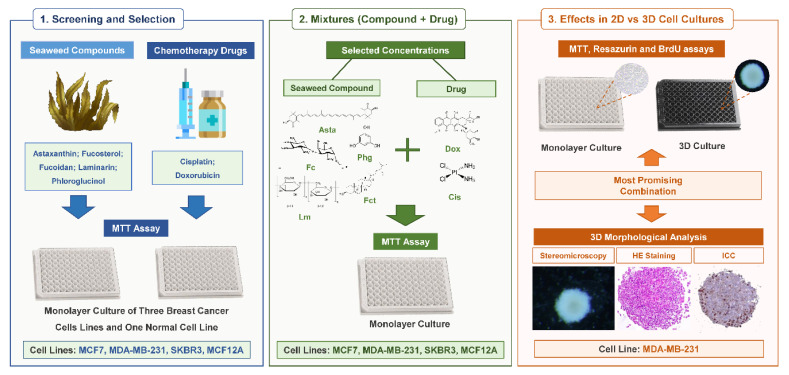
Schematic representation of the study design.

The study was performed in three phases according to a consecutive set of experiments (Figure 1). In Phase 1, it was performed a screening of the cytotoxic effects of five selected bioactive seaweed compounds (Asta, Fc, Fct, Lm, and Phg) and the two reference drugs (Cis and Dox). Each compound and drug were tested at five concentrations (Table 1). In Phase 2, two concentrations of each compound used in Phase 1 were selected to be tested in combination (seaweed compound + reference drug). The selected combinations are described in Table 2. In Phases 1 and 2, the screenings were performed in the panel of breast cell lines (MCF7, SKBR3, MDA-MB-231, and MCF12A), cultured in monolayer, and the cytotoxic effects were assessed by MTT assay. The most promising combination obtained in Phase 2 moved to Phase 3. That combination corresponded to the mixture that presented not only a statistically significant cytotoxic effect but also the highest % of reduction of cell viability when compared with the respective isolated compounds. Additionally, higher concentrations of the reference drug were introduced in Phase 3 to guarantee there was a positive control with cytotoxic effects in 3D. At Phase 3, other assays besides MTT were performed: resazurin and BrdU assay for assessing cytotoxic and cell proliferation effects, respectively. Also, the same conditions were tested simultaneously in monolayer (2D) and in 3D cultures for comparison purposes, and a morphological analysis of the 3D cultures was performed.

#### 2.3.2. Exposures (Single or Combination) in Monolayer

Cells were seeded at the density of 0.05 × 10^6^ cells/mL, 100 µL/well, and incubated for 24 h for cell attachment. Then the culture medium was removed, and cells were exposed to the tested conditions for 72 h. At the end of the exposure period, respective cell viability or proliferation assays were performed, according to the phase of the study. Tested concentrations of seaweed bioactive compounds and reference drugs used in the screening of Phase 1 are detailed in Table 1.

Prior to the exposures, the different solvent controls (medium; medium with 0.1% DMSO; and medium with 0.0009% NaCl) were tested in the four cell lines, using MTT to evaluate the effects on cell viability. Four independent methodological experiments (with triplicates per each experiment) were performed and no significant differences among solvents were found (data not shown). For this reason, we opted for using the most common one for the set of compounds—medium with 0.1% DMSO— as the solvent control in all experiments.

Regarding the selection of the concentrations to be tested in combination (Phase 2), we opted to use those that did not have a statistically significant effect on the cell viability of the tested cell lines or, in the case of the reference drugs, concentrations that did not reduce the cell viability below (in mean) 50%. These criteria were considered for each cell line. The selected combinations tested in Phase 2 are presented in Table 2.

#### 2.3.3. Exposures (Single or Combined) in 3D Cultures—Multicellular Aggregates (MCAs)

Cells were seeded in 96-well ultra-low attachment U-shaped spheroid plates (Corning, NY, EUA) at a density of 40 × 10^4^ cells/mL, 200 µL/well. Plates were then centrifuged in a centrifuge Rotina 380 R (Hettich, Vlotho, Germany) at 200× *g* for 10 min and placed in the incubator at 37 °C and 5% CO_2_ for 72 h to promote the MCAs formation. Then MCAs were incubated with the tested conditions for 96 h of exposure. After exposure, cell viability and proliferation assays as well as morphological analysis were performed.

### 2.4. Cell Viability Assessment

#### 2.4.1. MTT Assay

Cytotoxic effects of the tested conditions were assessed by MTT reduction assay. In short, 10 µL (monolayer) or 20 µL (3D) of MTT stock solution was added to each well, and incubated for 2 h (monolayer) and 4 h (3D), at 5% CO_2_ at 37 °C. At the end of the incubation period, MCAs must be transferred from the 96-well ULA plates to flat-bottom 96-well plates with the help of a P1000 micropipette with a cut tip. Exposure medium was then aspirated, and formazan crystals were dissolved by adding 100 µL of DMSO:ethanol solution (1:1) (*v*/*v*) (monolayer) or 150 µL of DMSO (3D). Plates were left for 15 min under mild agitation to promote total formazan salt dissolution. Absorbance was measured at 570 nm in a microplate reader Multiskan GO (Thermo Fisher Scientific, Waltham, MA, USA). Results are expressed as percentage of cell viability and were calculated based on the absorbance ratio between treated conditions and the untreated control (cells incubated with culture medium with 0.1% (*v*/*v*) of DMSO). In both situations (tested condition and control), the absorbance of respective mediums without cells was subtracted in each situation to eliminate interferences related to the compounds or drugs.

#### 2.4.2. Resazurin Assay

For resazurin assay, 1 µL (monolayer) or 2 µL (3D) of stock resazurin was added to each well. Plates were incubated for 3 h (monolayer) and 4 h (3D), with 5% CO_2_ and at 37 °C. Similarly, to what was performed for the MTT assay, MCAs and respective mediums were transferred to flat-bottom 96-well plates. Fluorescence was then read using excitation wavelength at 560 nm and emission wavelength at 590 nm in the plate reader Synergy H1 (Biotek, Winooski, VT, USA). Results are expressed as a percentage of cell viability in relation to control and were calculated based on the fluorescence ratio between treated conditions and the untreated control (cells incubated with culture medium with 0.1% (*v*/*v*) of DMSO). In both situations (tested condition and control), the fluorescence of respective mediums without cells was subtracted in each situation to eliminate interferences related to the compounds or drugs.

### 2.5. Cell Proliferation Assessment—BrdU Assay

Effects on cell proliferation were evaluated by BrdU assay (Roche, Basel, Switzerland). For monolayer, the assay was performed according to the manufacturer’s instructions. Briefly, cells were labeled with BrdU at a final concentration of 10 μM/well and incubated for 4 h at 37 °C, 5% CO_2_. Labeling medium was removed, and cells were stored at 4 °C overnight. Following the protocol, cells were fixed, and DNA denatured by the adding of 100 µL of FixDenant reagent (30 min). After removing the FixDenant Reagent, BrdU incorporation into cellular DNA was detected with mouse anti-BrdU conjugated with peroxidase working solution (diluted 1:100) (100 µL/well) for 90 min. Wells were rinsed with washing solution and 100 µL of substrate solution was added, to perform photometric detection. After 25 min, absorbances were immediately measured at 370 nm in a microplate reader Multiskan GO (Thermo Fisher Scientific, Waltham, MA, USA).

For 3D culture some alterations were implemented. Briefly, after MCAs exposure, 100 µL of each well medium was removed, and then MCAs were incubated with 10 µL of BrdU labelling solution (final concentration = 10 µM BrdU) for 5 h, at 37 °C, 5% CO_2_. Then, MCAs were transferred into a flat-bottom 96-well microplate and following the removal of the labelling medium, they were kept overnight at 4 °C. The following steps are identical to the monolayer protocol.

Results are expressed as a percentage of cell proliferation in relation to the control and were calculated based on the absorbance ratio between treated conditions and the untreated control (cells incubated with culture medium with 0.1% (*v*/*v*) of DMSO).

### 2.6. Cell Morphology Assessment

#### 2.6.1. Monolayer

The plates containing the monolayer cultures were observed photographed using a phase-contrast inverted microscope CX41 (Olympus, Tokyo, Japan).

#### 2.6.2. 3D–MCA Measurements

A total of 16 MCAs per tested condition/independent experiment were photographed at the end of the exposure time, using a stereomicroscope with darkfield SZX10 (Olympus, Tokyo, Japan), equipped with a digital camera DP21 (Olympus, Tokyo, Japan). The MCA areas were analyzed using the freeware AnaSP [101].

#### 2.6.3. Histological Analysis

##### MCA Histological Processing

At the end of exposure time, MCAs were collected to Eppendorf tubes with 10% buffered formalin (Bioptica, Milan, Italy) for fixation (24 h). For histological processing, MCAs were embedded in histogel (Thermo Scientific, MA, USA), according to manufacturer’s instructions. The following processing protocol consisted in dehydration-1 h in a crescent series of ethanol (70%, 90%, 95%, and two absolute); clearing-1 h in each reagent: xylene: absolute ethanol (1:1); xylene (twice); and infiltrating in liquid paraffin (1 h twice). Paraffin blocks were obtained in an embedding station EG 1140H (Leica, Nussloch Germany). Sections (3 µm) were performed in a microtome RM2255 (Leica, Nussloch Germany) and placed onto silane treated KP-frost slides (Klinipath, Duiven, The Netherlands). Slides were placed at 60 °C for 20 min, and then kept overnight at 37 °C.

##### Hematoxylin-Eosin (HE) Staining

Sections were deparaffinized in xylene and hydrated following the descendent sequence of ethanol (absolute, 95%, 70%), running tap water, 5 min each. Nuclei were stained with Mayer’s hematoxylin (Merck, Darmstadt Germany) for 3 min, and then slides were washed to remove dye excess. Following the protocol, sections were stained with 1% eosin Y for 5 min (Merck, Darmstadt, Germany). Lastly, slides were dehydrated in ethanol (95%, 100%, 100%), 5 min each, cleared in xylene (2 × 5 min), and mounted with the medium Q Path^®^ Coverquick 2000 (VWR Chemicals, Briari, France).

##### Immunocytochemistry (ICC)

For ICC, sections were deparaffinized and hydrated as described above. Heat antigen retrieval was made by sections immersion in citrate buffer 0.01 M, pH 6.0, using a pressure cooker (3 min after reaching maximum pressure). Slides were then allowed to cool, and then endogenous peroxidase blocking was performed with 3% hydrogen peroxide in methanol (10 min). After two washes in Tris saline buffer pH 7.6 (TBS) (5 min each), unspecific reactions were blocked using the appropriate reagent of the kit Novolink™ Polymer detection (Leica Biosystems, Milton Keynes, UK) (5 min), followed by two washes in TBST (TBS with 0.05% of Tween 20). Primary antibodies were diluted in phosphate buffer saline with 5% of bovine albumin serum and incubated 2 h at room temperature. For negative control, the primary antibody was substituted by the antibody diluent solvent only. Two primary antibodies were applied: rabbit monoclonal anti-Ki67, clone SP6 (Biocare Medical, USA) as cell proliferation marker [102,103], dilution 1:200, and rabbit polyclonal anti-caspase-3, ab 13,847 (Abcam, UK), diluted 1:5000 for assessing caspase dependent apoptosis [104,105]. Novolink™ Polymer detection system was used for signal amplification and revelation, according to manufacturer’s instructions, using the chromogen 3,3′-Diaminobenzidine (DAB). Nuclear counterstain was obtained using Mayer’s hematoxylin (2 min). Lastly, slides were washed, dehydrated, and mounted, then photographed as described before.

### 2.7. Statistical Analysis

Descriptive and inferential statistics were performed using Past3 (version 3.19) free software (https://folk.universitetetioslo.no/ohammer/past/) and GraphPad Prism 6.0 software (GraphPad Software, La Jolla, CA, USA). The normality and homogeneity of variance were confirmed by the Shapiro–Wilk and the Levene tests, respectively. The results are expressed as mean + standard deviation, except for the MCAs areas that were presented in median, maximum, minimum, and interquartile range (Q3–Q1), from at least five to six independent experiments (triplicate per each experiment). Significant differences (*p* < 0.05) were assessed by one-way ANOVA, followed by the post-hoc Holm–Šídák multiple comparison test. On selected cases, the significance of the difference between two groups of interest was tested with the Student’s *t*-test and using the sequential Holm–Bonferroni correction; the latter was implemented via a freeware spreadsheet calculator [106,107].

## 3. Results

### 3.1. Cytotoxic Effect of Seaweed Bioactive Compounds

**Figure 2:**
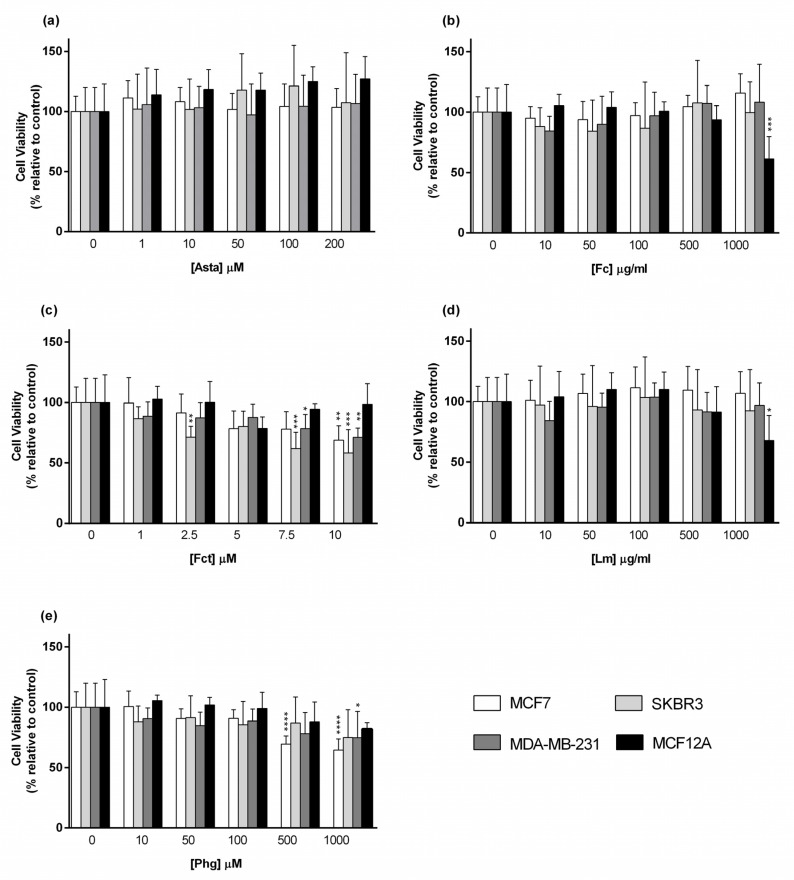
Cytotoxic effect of (**a**) Astaxanthin (Asta), (**b**) Fucoidan (Fc), (**c**) Fucosterol (Fct), (**d**) Laminarin (Lm), and (**e**) Phloroglucinol (Phg) assessed by MTT assay after 72 h of exposure in the panel of breast cell lines cultured in monolayer. Control corresponds to cells with medium containing 0.1% DMSO. The percentages of cell viability are relative to the controls and presented as mean + standard deviation of six independent experiments (each in triplicate). (* *p* < 0.05, ** *p* < 0.01, *** *p* < 0.001, **** *p* < 0.0001).

Cytotoxic effects of seaweed compounds were assessed by the MTT assay after 72 h of incubation in cultured monolayers. Figure 2 shows the results obtained for tested compounds in cellular viability. Asta was the only compound that had no effects on the cell viability in all used cell lines (Figure 2a). The polysaccharides Fc and Lm promoted a similar result since both only had cytotoxic effects in the non-tumoral cell line MCF-12A at the highest concentration (1000 µg/mL) (Figure 2b,d). Fct was the compound with more cytotoxic effects. It significantly decreased cell viability in SKBR3 (at 2.5 µM),and in SKBR3 and MDA-MB-231 cell lines (at 7.5 µM). At 10 µM it also decreased the cellular viability of the other BC cell line MCF7, however not affecting MCF12A cell line (Figure 2c). Phg decreased cell viability in MCF7 (at 500 µM and 1000 µM) and in MDA-MB-231 cell lines (at 1000 µM) (Figure 2e).

### 3.2. Cytotoxic Effect of the Reference Drugs–Cisplatin and Doxorubicin

Five crescent concentrations were used to assess the cytotoxic effects of Dox and Cis in the panel of breast cell lines, and the effects on cell viability were assessed by the MTT assay.

**Figure 3:**
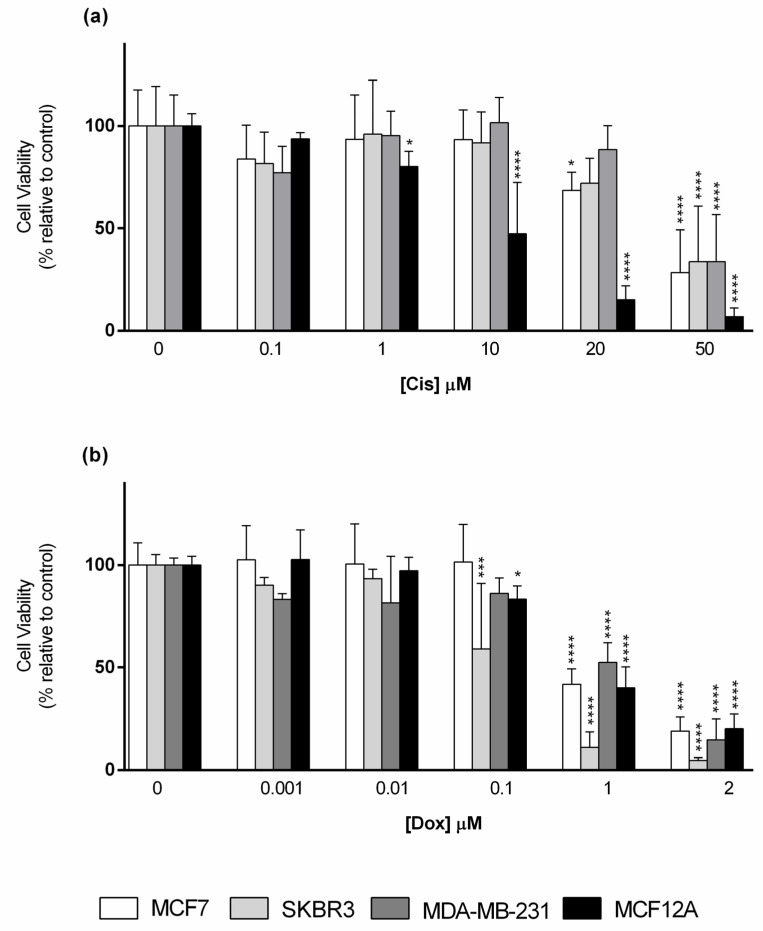
Cytotoxic effect of (**a**) Cisplatin (Cis); (**b**) Doxorubicin (Dox) assessed by the MTT assay after 72 h of exposure in the panel of breast cell lines cultured in monolayer. Control corresponds to cells incubated with medium containing 0.1% DMSO. The percentages of cell viability are relative to the controls and presented as mean + standard deviation of six independent experiments (each in triplicate). (* *p* < 0.05, *** *p* < 0.001, **** *p* < 0.0001).

Considering Cis exposure (Figure 3a), the non-tumoral cell line (MCF12A) was the most susceptible to this drug, being the only cell line that showed a statistically significant reduction on cell viability when cells were exposed to Cis at 1 µM, inducing then a concentration-dependent response. In contrast, MCF7, only showed significant differences in cell viability at Cis (20 µM and 50 µM), while SKBR3 and MDA-MB-231 were still more refractive to Cis action, with a lowering trend at 20 µM that reached significance at 50 µM. At the latter concentration, all cell lines had their cell viability decreased below 50%, in relation to the control.

In relation to Dox cytotoxicity, this drug started to significantly reduce the viability of SKBR3 and MCF12A at 0.1 µM, while for the other two cell lines this effect was only observed at Dox 1 µM and 2 µM. At 1 µM, a reduction in cell viability below 50%, in relation to the control, was observed in all cell lines (Figure 3b).

### 3.3. Cytotoxic Effect of Selected Combinations of Seaweed Bioactive Compound Plus Reference Drug

In vitro cytotoxic effects of the five seaweed compounds combined with the two reference drugs were assessed by the MTT assay in the panel of BC cell lines. For that, two concentrations of each seaweed compound and two concentrations of each drug were selected for the combination according to the criteria mentioned in Section 2.3.2.

**Figure 4:**
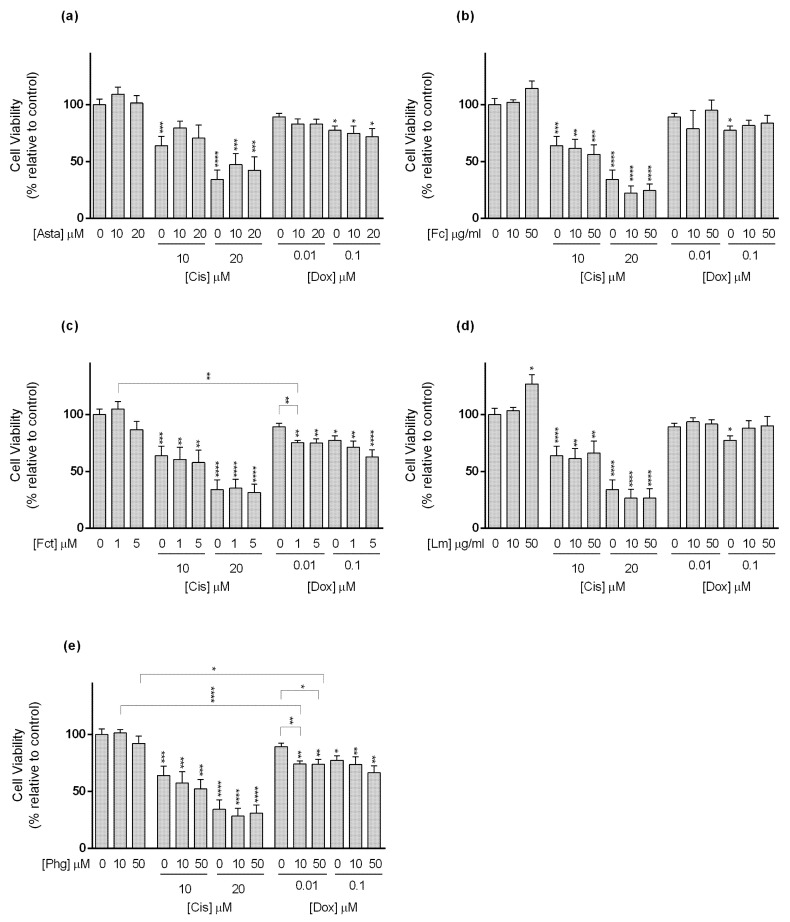
Cytotoxic effects of the combination of (**a**) Astaxanthin (Asta); (**b**) Fucoidan (Fc); (**c**) Fucosterol (Fct); (**d**) Laminarin (Lm); (**e**) Phloroglucinol (Phg) with the reference drugs cisplatin (Cis) and doxorubicin (Dox) assessed by the MTT the assay after 72 h of exposure in MCF7 cell line cultured in monolayer. Control corresponds to cells incubated with medium containing 0.1% DMSO. The percentages of cell viability are relative to the control and presented as mean + standard deviation of six independent experiments (each in triplicate). Square brackets indicate *t* tests with Sequential Bonferroni corrections. (* *p* < 0.05, ** *p* < 0.01; *** *p* < 0.001, **** *p* < 0.0001).

For MCF7 cell line (Figure 4), Cis alone at tested conditions (10 and 20 µM) decreased cell viability in a concentration-dependent manner. Among the tested seaweed compounds, only Asta was able to reduce the effect of Cis. This happened when cells were exposed to Cis (10 µM) in combination with Asta (10 and 20 µM) (Figure 4a).

Still in MCF7 cells, only Dox at 0.1 µM significantly decreased cell viability. Nevertheless, Dox (0.01 µM) alone did not show effects on cell viability, but when combined with Fct (1 and 5 µM), and Phg (10 and 50 µM) (Figure 4c,e, respectively), it significantly affected MCF7 cells’ viability. Dox (0.01 µM) did not differ from the control, but in combination, cell viability decreased by ≈15% when compared to the drug alone, differing from the control. However, in the case of the combination of Dox (0.01 µM) plus Fct (5 µM), viability did not differ from the Fct alone. In the case of Fc and Lm in combination with Dox (0.1 µM), the effect was the opposite, and the combination decreased the cytotoxic effect induced by the Dox alone (Figure 4b,d). In line with this result, cells exposed to Lm (50 µM) presented significantly higher cell viability than the control (Figure 4d).

**Figure 5:**
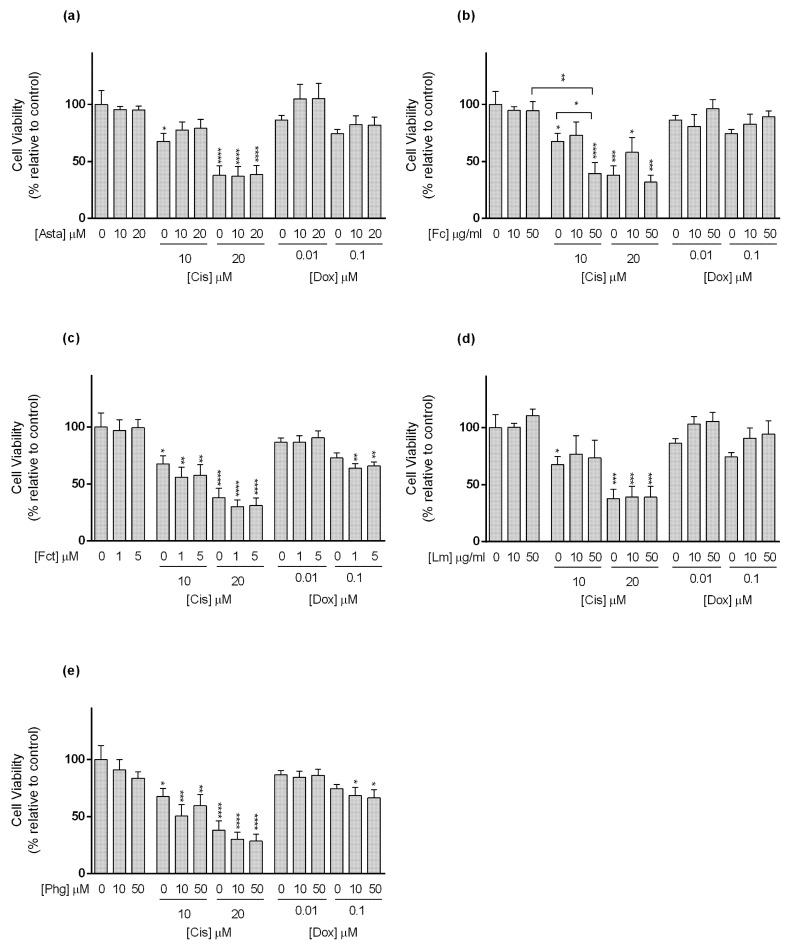
Cytotoxic effects of the combination of (**a**) Astaxanthin (Asta); (**b**) Fucoidan (Fc); (**c**) Fucosterol (Fct); (**d**) Laminarin (Lm); (**e**) Phloroglucinol (Phg) with the reference drugs cisplatin (Cis) and doxorubicin (Dox) assessed by the MTT assay after 72 h of exposure in SKBR3 cell line cultured in monolayer. Control corresponds to cells incubated with medium containing 0.1% DMSO. The percentages of cell viability are relative to the control and presented as mean + standard deviation of six independent experiments (each in triplicate). Square brackets indicate *t* tests with Sequential Bonferroni corrections. (* *p* < 0.05, ** *p* < 0.01; *** *p* < 0.001, **** *p* < 0.0001).

Regarding the SKBR3 line (Figure 5), Cis alone reduced cell viability at the tested concentrations. As to the combinations, the following conditions: Cis (10 µM) plus Asta (10 and 20 µM), Fc (10 µM), and Lm (10 and 50 µM), decreased the cytotoxicity of the drug not differing from the control. However, the combination Cis (10 µM) with Fc (50 µM) decreased cell viability to a percentage that statistically differed from the drug and Fc alone (Figure 5b). This combination enhanced the cytotoxic effect of Cis in ≈28%. Nonetheless, in the combination of Dox at 0.1 µM with Fct (1 and 5 µM), and Phg (10 and 50 µM), the cell viability differed from the control, not differing from the drug nor the compound alone (Figure 5c,e).

**Figure 6:**
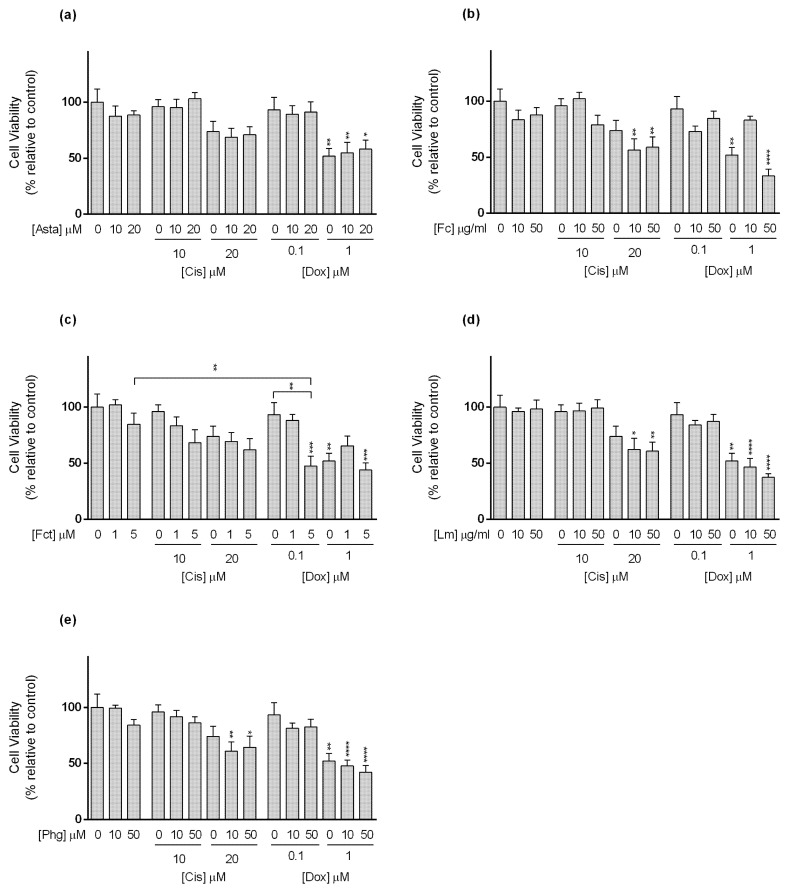
Cytotoxic effects of the combination of (**a**) Astaxanthin (Asta); (**b**) Fucoidan (Fc); (**c**) Fucosterol (Fct); (**d**) Laminarin (Lm); (**e**) Phloroglucinol (Phg) with the reference drugs cisplatin (Cis) and doxorubicin (Dox) assessed by the MTT assay after 72 h of exposure in MDA-MB-231 cell line cultured in monolayer. Control corresponds to cells incubated with medium containing 0.1% DMSO. The percentages of cell viability are relative to the control and presented as mean + standard deviation of six independent experiments (each in triplicate). Square brackets indicate *t* tests with Sequential Bonferroni corrections. (* *p* < 0.05, ** *p* < 0.01; *** *p* < 0.001, **** *p* < 0.0001).

With reference to the TNBC cell line MDA-MB-231 (Figure 6), Cis (10 and 20 µM) alone did not present significant differences in cell viability in relation to the control. Conversely, when in the following combinations Cis 20 µM plus Fc (10 and 50 µg/mL), Lm (10 and 50 µg/mL), and Phg (10 and 50 µM), cell viability significantly decreased relative to control, with a reduction between 13 and 17%, but did not differ statistically from the drug alone (Figure 6b,d,e).

In relation to Dox, alone at 1 µM, it negatively affected cell viability. When combined with Fc (10 µg/mL) and Fct (1 µM), Dox at 1 µM seemed to have lost its action, while in combination with Fc (50 µg/mL) and Fct (5 µM) its effects were maintained (Figure 6b,c). The combination differing from control and with the most evident impact in cell viability, when compared to either to the compound or to the drug alone, was Dox (0.01 µM) with Fct (5 µM), which increased Dox cytotoxicity in ≈46%.

**Figure 7:**
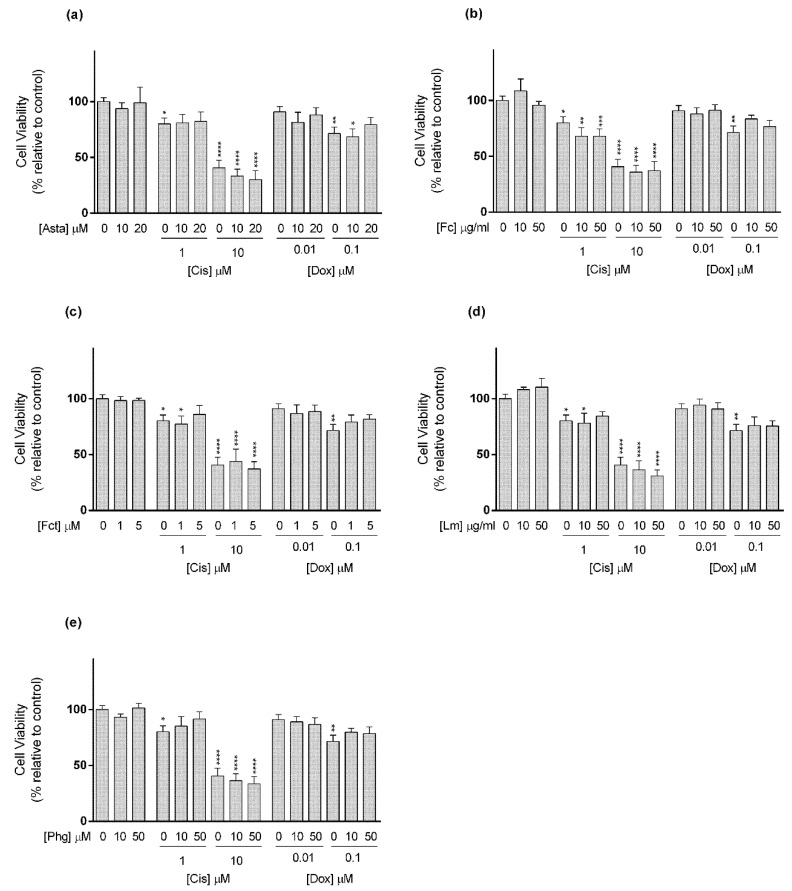
Cytotoxic effects of the combination of (**a**) Astaxanthin (Asta); (**b**) Fucoidan (Fc); (**c**) Fucosterol (Fct); (**d**) Laminarin (Lm); (**e**) Phloroglucinol (Phg) with the reference drugs cisplatin (Cis) and doxorubicin (Dox) assessed by the MTT assay after 72 h of exposure in MCF12A cell line cultured in monolayer. Control corresponds to cells incubated with medium containing 0.1% DMSO. The percentages of cell viability are relative to the control and presented as mean + standard deviation of six independent experiments (each in triplicate). (* *p* < 0.05, ** *p* < 0.01; *** *p* < 0.001, **** *p* < 0.0001).

In relation to MCF12A cell line (Figure 7), Cis (1 and 10 µM) alone decreased cell viability in relation to control. The combination of Cis 1 µM with Asta (10 and 20 µM), Fct (5 µM), Lm (50 µg/mL), and Phg (10 and 50 µM), caused the loss of statistical significance found in the drug alone. At 10 µM, Cis alone and all the combinations showed cell viability of less than 50% in relation to the control. Regarding Dox, only the highest tested concentration (0.1 µM) showed a significant effect on cell viability, however, in this cell line, it occurred the loss of statistical effect in combination with all tested seaweed compounds (Figure 7).

The results of the combinations in the panel of cell lines are summarized in Table 3, using a color code to discriminate the differences in relation to the control.

### 3.4. Comparative Study of One Promising Combination—Monolayer vs. 3D Cultures

For comparison purposes, we selected the most promising combination in which the seaweed compound and the drug alone did not have any effect on cell viability in relation to the control, but the combination potentiated the effect of the drug, that is, differing from the control and from the compound and drug alone. The selected combination was Fct 5 µM with Dox 0.1 µM in MDA-MB-231 cell line, as it revealed the most evident effect on cell viability, showing, on average, 46% less cellular viability than the drug alone. This selected combination was tested simultaneously in monolayer and in 3D culture, the latter providing multicellular aggregates (MCAs). Considering that 3D cultures are commonly more resistant to treatments [50,108], we augmented the concentration of Dox (1, 2, and 5 µM) to allow the visualization of the drug effect and to have a concentration that served as a positive control (in this case Dox 5 µM). An all-new set of experiments was conducted, with new replicas, where cell viability was assessed by MTT and resazurin assays, and cell proliferation was evaluated by the BrdU assay. Additionally, MCAs were evaluated by performing area measurement, histological and immunocytochemical analysis.

#### 3.4.1. MTT Assay

**Figure 8:**
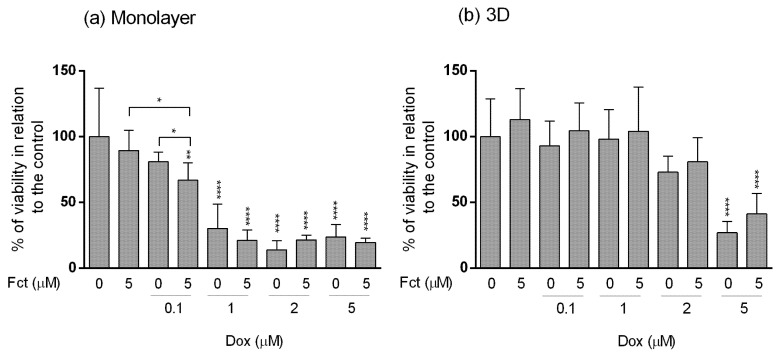
Effect of fucosterol (Fct) at 5 µM alone and in combination with doxorubicin (Dox) at 0.1, 1, 2 and 5 µM, on the viability of MDA-MB-231 cells in monolayer–72 h (**a**) and 3D–96 h (**b**) assessed by the MTT assay. Cells treated with 0.1% DMSO and Dox 5 µM were included as negative and positive controls, respectively. The percentages of cell viability are relative to the control and presented as mean + standard deviation of five independent experiments (each in triplicate). (* *p* < 0.05, ** *p* < 0.01 and **** *p* < 0.0001).

The results obtained in monolayer by the MTT assay (Figure 8a) were very comparable to those presented before in Section 3.3. Fct alone did not present effects on cell viability. Dox (≥1 µM) showed high cytotoxicity, with cell viabilities under 50% in relation to the control. The selected combination of Fct (5 µM) with Dox (0.1 µM) statistically differed from the control and from drug and seaweed compound alone. As for the combination with higher Dox concentration, there were no statistical differences when compared to drug alone.

In 3D culture (Figure 8b), the results were very different from the ones obtained in monolayer. The cell viability in 3D only differed from the control when cells were exposed to Dox at 5 µM, alone or in combination with Fct (5 µM).

#### 3.4.2. Resazurin Assay

**Figure 9:**
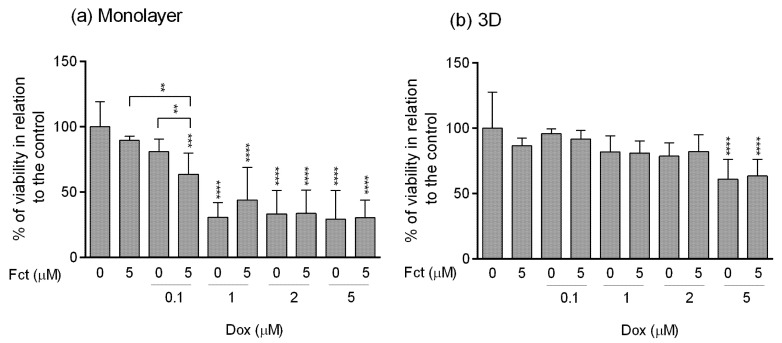
Effect of fucosterol (Fct) at 5 µM alone and in combination with doxorubicin (Dox) at 0.1, 1, 2, and 5 µM, on the viability of MDA-MB-231 cells in monolayer–72 h (**a**) and 3D–96 h (**b**) assessed by the resazurin assay. Cells treated with 0.1% DMSO and Dox 5 µM were included as negative and positive controls, respectively. The percentages of cell viability are relative to the control and presented as mean + standard deviation of five independent experiments (each in triplicate). Square brackets indicate *t* tests with Sequential Bonferroni corrections (** *p* < 0.01, *** *p* < 0.001 and **** *p* < 0.0001).

The resazurin assay performed in monolayer (Figure 9a) reproduced the results obtained in the MTT assay. Fct alone did not impact cell viability, while all Dox (≥1 µM) conditions significantly differed in cell viability relative to the control. Fct (5 µM) and Dox (0.1 µM), alone, did not differ from the control, however, the combination Fct (5 µM) plus Dox (0.1 µM) significantly decreased cell viability relative to control and to the seaweed compound alone.

Also, as in the MTT assay, cells in 3D culture (Figure 9b) were more resistant to drug treatment, only revealing significant cytotoxic effect in cells exposed to Dox (5 µM), alone and in combination with Fct (5 µM).

#### 3.4.3. Assessment of Cell Proliferation

**Figure 10:**
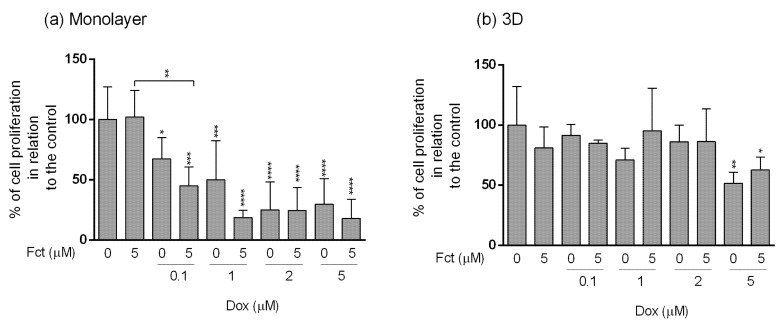
Effect of fucosterol (Fct) at 5 µM alone and in combination with doxorubicin (Dox) at 0.1, 1, 2, and 5 µM, on cell proliferation in monolayer–72 h (**a**) and 3D–96 h (**b**) assessed by BrdU assay. Cells treated with 0.1% DMSO and Dox 5 µM were included as negative and positive controls, respectively. The percentages of cell proliferation are relative to the control and presented as mean + standard deviation of five independent experiments (each in triplicate). (* *p* < 0.05, ** *p* < 0.01; *** *p* < 0.001; **** *p* < 0.0001).

MDA-MB-231 cells cultivated in monolayer (Figure 10a) showed a decrease in cell proliferation comparatively to the control, in all Dox concentrations (from 0.1–5 µM), and also in all combinations with Fct (5 µM). The combination Dox (0.1 µM) with Fct (5 µM) differed from the control and from the Fct alone, but did not differ from the drug alone. Although no significant statistical differences in cell proliferation were detected, graphically it seems that the combination of Dox (0.1 and 1 µM) with Fct (5 µM) had more effect than the drug alone (decreasing the mean of cell proliferation in 22 and 31%, respectively). Fct alone did not have any effect on cell proliferation.

The effects on cell proliferation in 3D culture (Figure 10b) followed the same tendency as the viability assays, showing more resistance to the treatments. There were significant differences only in cells exposed to Dox (5 µM) alone (positive control) or in combination with Fct.

#### 3.4.4. Morphological Analysis of 3D Cultures (MCAs)

##### MCAs Measurements

**Figure 11:**
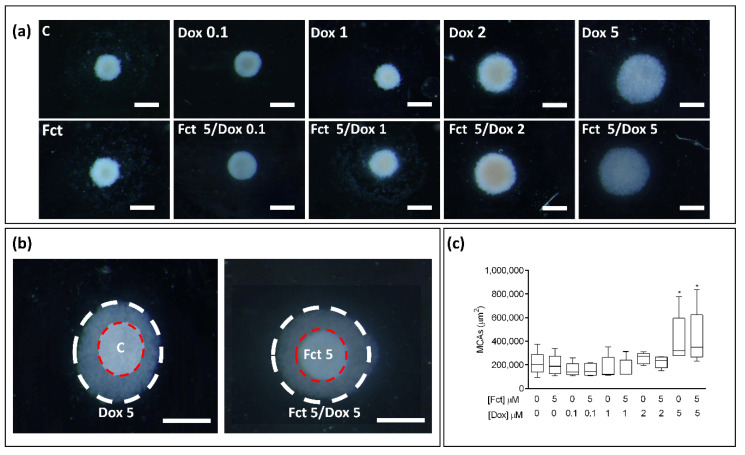
Representative stereomicroscopic images of 3D cultures-MCAs in the tested conditions of fucosterol (Fct) at 5 µM alone, and in combination with doxorubicin (Dox) at 0.1, 1, 2, and 5 µM. Cells treated with 0.1% DMSO (C) and Dox (5 µM) were included as negative and positive controls, respectively (**a**). Two images of MCAs from C and Fct (5 µM) (red dashed circle) and Dox (5 µM) and Fct (5 µM) + Dox (5 µM) (white dashed circle) are overlapped to show the difference in cellular aggregation between the two tested conditions (**b**). Box and whisker graph of Areas of MCAs expressed as median, maximum, minimum, and interquartile range (Q3-Q1 of five independent experiments (16 MCAs/per tested condition/per experiment) (**c**). Significant differences: * *p* < 0.05. Scale bar: 500 µm.

In the stereo microscopic observation of the MCAs (Figure 11a), those exposed to Dox (2 and 5 µM) (alone and in combination), revealed a loosening effect, which was much more evident in the conditions with Dox (5 µM). In Figure 11b, representative images of MCAs control (C) and Dox (5 µM), Fct (5 µM) and Fct/Dox (both 5 µM), photographed at the same magnification, were overlapped to highlight this loosening effect. In both situations, there is an evident loosening of the MCAs. There were no differences between the MCAs exposed to the drug alone and its respective combination with Fct (5 µm). The MCAs photographs were analyzed using AnaSP software. The determined areas are presented in Figure 11c, where it is possible to observe that besides the visual impression from stereomicroscopy, only the conditions with Dox at 5 µM significantly differed from the control.

##### Histological and Immunocytochemical Analysis

**Figure 12:**
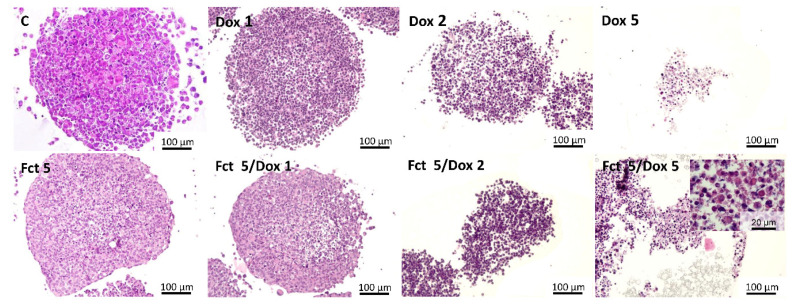
Representative histological images of MCAs exposed to the tested conditions: fucosterol (Fct) 5 µM alone and in combination with doxorubicin (Dox) 1, 2, and 5 µM. Cells treated with 0.1% DMSO correspond to the control (C). MCAs sections were stained with hematoxylin-eosin.

After 96 h of exposure, MCAs were fixed, processed for paraffin embedding, and sectioned for hematoxylin-eosin (HE) staining and immunocytochemistry (ICC) analysis. By observing the MCAs stained with HE, the combinations in which morphological alterations were present are given in Figure 12. Under Dox (1 µM) (alone and combined) the alterations were very subtle, a higher number of cells with hyperchromatic and pyknotic nuclei were observed, but the MCAs structure was intact. Differently, in the MCAs exposed to Dox (2 and 5 µM) (alone and in combination with Fct), the structure of the MCAs was concentration-dependent damaged, with looser structure, where cells lost their attachment, and with an increased number of cells with death compatible morphology, as shown in higher magnification in the inserted image of Fct/Dox 5 combination (at the bottom rigth of Figure 12). MCAs exposed to Dox (5 µM) tended to disintegrate quickly, forming a cell suspension. Morphologically, there was no difference between the MCAs exposed to Dox alone and the respective combination with the Fct (5 µM). No necrotic core was observed in the sectioned MCAs.

**Figure 13:**
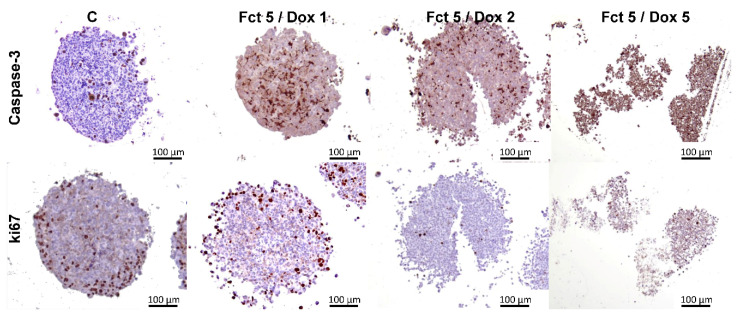
Representative histological images of MCAs immunostained against caspase-3 and ki67 after exposure to fucosterol (Fct) at 5 µM in combination with doxorubicin (Dox) at 1, 2 and 5 µM. Cells treated with 0.1% DMSO correspond to the control (C). Brown color-diaminobenzidine (DAB) indicates positive staining.

Antibodies against caspase-3 and ki67 were used for ICC. Here we show representative images of the control (C) and the combinations of Fct with Dox (1, 2, and 5 µM), as these were the conditions in which visual alterations of ICC staining existed in relation to the control (Figure 13). Also, in each drug concentration, the results were very similar between the drug alone and its combination with Fct. The outcomes showed that there were caspase-3 positive cells in the MCAs of all tested groups, including in the control, being these positive cells randomly distributed throughout all the MCAs. However, the number of stained cells in the C group is much lower when compared with the positive cellularity in the drug-exposed groups. Positive caspase-3 cells in the groups exposed to Dox (1 and 2 µM) were similar, but, when using Dox (5 µM), more than 80% of all cells were positive, indicating a high degree of cell death.

In relation to the immunostainings for ki67, positive cells were also distributed all along the MCAs, with more predominance in their outer region (Figure 13). In Dox (0.1 and 1 µM) groups, the number of Ki67 positive cells seemed similar to the control. When it comes to MCAs of the Dox groups (2 and 5 µM), and its combination with Fct, the number of positive cells were visibly lower; less than 10% of the total number of cells (Figure 13).

## 4. Discussion

This study explored the cytotoxic effects of five brown seaweed compounds alone and combined with two reference drugs in a panel of breast cell lines, representing three BC subtypes and including a non-tumoral breast cell line. The study is justified considering that seaweed compounds, especially those from brown seaweeds, have been showing anticarcinogenic activities in many in vitro and in vivo studies related to many types of cancers, including BC [22,24,54,109,110]. The effects of combining seaweed compounds plus chemotherapeutic drugs are also relevant to explore, taking into consideration their implications in clinical scenarios. Despite their importance, the literature about this topic is still scarce [37,38,39,73,111]. Several studies with natural products, mostly in vitro, described beneficial combinatory effects with several anticancer drugs in BC, through diverse action mechanisms, suggesting that these combinations represent a promising strategy to treat BC [95,112]. However, in a clinical scenario, interactions can occur, potentially affecting drug effects [113]. In this vein, many seaweed compounds have antioxidant properties, and the intake of antioxidants during chemotherapy is very controversial, requiring further studies [28,29,114,115]. In connection with this problem, there is nowadays easy access in classical herbalists, or on the internet, to commercially available seaweed products without a medical doctor’s prescription and appropriate legislation.

In this study, we started screening five seaweed compounds and two selected drugs in a panel of four breast cell lines, testing five concentrations of each, and then selected two of them for the combinations, according to pre-established criteria. Although there are some data related to the effects of the drugs in the used cell lines, the IC_50_ values vary from study to study, from 2- to 10-fold of concentration within the same line [116,117,118]. For this reason, we preferred to screen and select a drug concentration based on our cell culture conditions. In the end, we chose the most interesting result of the combinations in monolayer and tested it in a more complex 3D in vitro model [50,119].

Regarding the cytotoxicity of the carotenoid Asta, alone (1–200 μM), it had no have effects on cell viability. This contrasts with previous studies that reported that Asta (50 μM) induced apoptosis in T-47D and MDA-MB-231 cells (both BC cell lines) [120], reduced proliferation rates and inhibited cell migration in MCF7 and MDA-MB-231 cell lines [62]. When in combination with Cis, in MCF7, SKBR3, and MCF12A cell lines, Asta interfered with this drug action, as in the mixture, Cis 10 μM lost its effect. Asta has been previously described to confer protection against oxidative stress [121] and, in in vivo studies with rats, it had a protective effect against Cis-induced toxicity in the gastrointestinal tract [122], ear [123], and also retina [124], which could partially explain the loss of effect observed in the present study.

In relation to the polysaccharides, Fc and Lm alone caused significant cytotoxicity in MCF12A cell line at the highest tested concentration (1000 μg/mL). In contrast, other authors reported that Fc from 300 to 1000 μg/mL decreased cellular viability in a dose-dependent manner, induced G1 phase arrest, promoted ROS induction and triggered apoptosis through caspases-dependent pathway [125]. Another work described that Fc at 400 μg/mL inhibited cell proliferation measured by the MTT assay, in MDA-MB-231 and MCF-7 cells [73]. It is unclear why there is such inter-study variability, particularly when using the same cell line, but the facts warn for caution regarding accepting definitive conclusions.

In combination, Fc modelled different effects according to the drug and the cell line tested. In SKBR3, Fc (10 μg/mL) in combination with Cis (10 μM) decreased Cis cytotoxicity, while the combination with Fc (50 μg/mL) statistically increased cell toxicity, differing from both the compound and the drug alone. A similar pattern of Cis enhancement effect with Fc was observed in MCF7, MDA-MB-231, and MCF12A cell lines, even if not with the same statistical significance, suggesting that Fc in higher concentrations may potentiate the effect of Cis in all cell lines. These findings are in line with previous studies where Fc significantly enhanced the cytotoxicity of Cis, Dox, and taxol in MCF7 cells [126]. Fc at 400 μg/mL in co-treatment with Cis at 5 and 10 μM, enhanced intracellular ROS and reduced glutathione (GSH) levels in MCF7 and MDA-MB-231 cells, suggesting that the induction of oxidative stress was an important event in the cell death induced by the combination in those BC cell lines [73].

In some combinations of Fc with Dox—Fc at 10, 50 μg/mL with Dox at 0.1 μM, in MCF7 and MCF12A; Fc at 10 μg/mL with Dox at 1 μM, in MDA-MB-231—Fc seems to decrease Dox effect compared to Dox alone, since in the latter case Dox significantly differed from the control. In contrast, this effect was not verified in the referred combinations. Our results are in contradiction with a previous study describing that Fc enhanced Dox effects [126]. In the opposite view, in vivo and in vitro studies in rodent models suggest that Fc may play a protective role in Dox-induced acute cardiotoxicity [127]. Therefore, in our research and in some conditions, Fc may have protected the tested breast cell lines from Dox cytotoxicity. These results indicate that we are far from understanding, controlling, and predicting the effects of Fc over cancer cells. In this sense, the anticancer activities of fucoidans continue to be explored, and recently these compounds have been used in clinical trials to evaluate their potential synergy with other anticancer therapies, in several cancer types, including BC [128].

The other tested polysaccharide, Lm, when tested alone, presented significant higher cellular viability than the control in MCF7. Although not statistically significant, the same tendency was observed in SKBR3 and MCF12A cells. Hypothetically, Lm can protect cells from oxidative stress produced by cellular metabolism. Treatment of mouse thymocytes with Lm suppressed apoptotic death around 2- to 3-fold and extended cell culture survival in about 20–30% [129]. Our study in BC cells is well in line with the hypothesis and the given proof of concept with thymocytes. However, a study reported the cytotoxic effects of Lm at 200 μg/mL in MDA-MB-231 cell line [130], and another one described a reduction in cell viability in MCF7 and MDA-MB-231 cell lines exposed to Lm from 12.5 to 400 μg/mL [81].

As to the combinations with Cis, this drug lost its effects in some combinations with Lm (Lm at 10, 50 μg/mL in SKBR3 and Lm at 50 μg/mL in MCF12A). Conversely, in MDA-MB-231, Cis (20 μM) alone did not influence cell viability, but when in combination with Lm it presented lower cell viability, differing from the control. The literature related to the combination of Lm with Cis and Dox is very scarce, but it has been already described a protective effect against Cis-induced toxicity in auditory cells [131]. When combined with Dox, Lm inhibited its effect in MCF7 and MCF12A cells. To the best of our knowledge, no literature was found in relation to the combination of Lm and Dox.

Considering the phlorotannins, Pgh alone had a cytotoxic effect in MCF7 from the concentration of 500 μM, and in MDA-MB-231 at 1000 μM. Our data corroborate a prior study reporting that Pgh at 100 μM was not cytotoxic to MCF7 and SKBR3 cell lines [92]. At the latter concentration, Phg suppressed cell migration and invasion in MDA-MB-231 cell line [132]. At higher concentrations than those described here, Phg induced cytotoxicity through caspases activation in MDA-MB-231 cell line [91].

In combination with Cis, two different effects were observed: in MDA-MB-231, Pgh with Cis (20 μM) increased Cis cytotoxicity differing from the control, while in MCF12A, the combination with Cis (1 μM) negatively affected this drug action, rescuing cell viability to the control levels. Therefore, our results point to the possible protective and potentiating effects of Cis, depending on the cell line and the concentration used. The literature is also contradictory. On one hand, Pgh displayed a protective effect against Cis-induced cell death in normal human urothelial and bladder cancer cells [133]. On the other hand, the exposure with Phg before Cis treatment, sensitized the cell to this drug, enhancing its cytotoxic effect in BC cell lines [92]. Phg also enhanced the tumoricidal effect of Cis in ovarian cancer cells in a rodent model [134].

The combinations of Phg with Dox (0.01 μM) potentiated the effects of the drug in MCF7, where the combination differed from the control and both the compound and drug alone. A similar effect was observed with Dox (0.1 μM) in SKBR3, but did not differ from the compound and drug alone. However, in MCF12A, the opposite effect was observed. In the literature, we only found reports of the protective effects of Phg: cardioprotective agent against doxorubicin-induced cardiotoxicity [135]. Some new Pgh derivatives reversed multidrug resistance in Dox-induced resistant MCF7/Dox sublines [136]. This finding agrees with ours, that is suggestive of Dox potentiation against MCF7 and calls for more studies to refine what appears to be a promising interaction.

Due to the conflicting information in the literature in relation to the combination of seaweed compounds with anticancer drugs, more studies are necessary to elucidate both methodological aspects and mechanisms beyond these contradictory results. The differences between our study and several others in relation to the cytotoxicity of seaweed compounds can be justified by many possible reasons. First, some concentrations largely differ, and, as we observed, different concentrations can trigger different responses within the same model. Secondly, there are differences in the sources of compounds. We used high purity commercial compounds, but most of the other studies used extracts from different seaweeds species, with diverse extraction processing and certainly different degrees of purity that could interfere with the results. Also, for some compounds, such as Fc, the molecular weight was considered a critical factor for its anticancer activity, and different fucoidans can present different molecular weights [137]. Similarly, the hepatoprotective effects of phlorotannins against Dox-induced cytotoxicity were related to the molecular weight of phlorotannins [138]. Thus, for these cited compounds, and possibly for others, the specific chemical characteristics can determine the biological effect and their bioavailability. Beyond chemistry and experimental procedures, even the statistical analyses follow different options that influence the acceptance/rejection of hypotheses and conclusions.

From the five tested compounds, Fct had the most promising results. Fct alone at 10 μM induced cytotoxicity effects in the three BC cell lines, not affecting the non-tumoral cell line (MCF12A). Such pattern also existed in some other studies. Fct containing fractions of seaweeds were cytotoxic for colon cell lines and T47D BC cell line, without cytotoxic effects on the normal cell line [86]. Fct from lipid extracts of Antarctic seaweeds also reduced cell proliferation and induced apoptosis in MCF7 and MDA-MB-231 cell line, but these effects were not so evident in the non-tumoral cell line CHO [88].

Globally, and according to the present data and literature, Fct seemed to have less (or even no) impact on the non-tumoral cell lines than in cancer ones. This notion goes beyond breast cells. Indeed, besides colon cell lines [87], others reported that Fct exerted minimal cytotoxicity in non-tumoral lung cell lines with IC_50_ ˃ 100 μM [139], therefore suggesting that Fct selectively impacts cancer cells. Conversely, Fct did not show any cytotoxicity in the liver cancer cell line HepG2 at concentrations up to 100 μM [140].

For the Fct combination with drugs, we selected a non-cytotoxic concentration of the former. The combination of Fct with Cis did not differ from the effects of Cis alone. Contrary to our results, one study showed a synergistic anticancer effect of Fct in combination with Cis, related to the expression of apoptotic and angiogenic genes, in ovarian cancer cell lines [141].

Differently, the combination of Fct with Dox enhanced cytotoxicity in MCF7 and MDA-MB-231 cells. In both situations, the lowest Dox concentration did not have cytotoxicity alone. Still, combined with Fct, the cytotoxic effects increased, differing from the control and the compound and drug alone. In MDA-MB-231, the potentiation of Dox effects was more evident, because Dox (0.1 μM) in combination with Fct (5 μM) differed from the control, from Fct alone and almost doubled the impact of the drug alone. In SKBR3 the same enhancement trend of Dox toxicity was observed, but without statistical significance. By the opposite, in MCF12A cell line, the combination of Fct with Dox increased cell viability suggesting a protective effect against Dox action.

Notably, the most promising combination involving Fct was noted in the MDA-MB-231 cell line, representative of TNBC. From our data, Fct seems to be a promising drug adjuvant in this type of BC type. Owing to the lack of ER, PR, and HER2 receptors, which are nowadays the available BC target therapies, TNBC presents poor prognosis, being the systemic chemotherapy (often using Dox) the mainstream treatment [142,143,144]. Despite the advancement of molecular technologies that identified TNBC as a disease with intrinsic molecular and immunological heterogeneity, recognizing the variety of clinical phenotypes and revealing several putative biomarkers in TNBC, some of them already used in clinical approach, there is necessary preclinical and clinical research mainly for resistant population in order to improve the development of new therapeutic strategies [145,146]. Drug resistance is also a major problem in the treatment of TNBC [147]. Thus, the investigation of new drugs or drug adjuvants to boost cytotoxicity, overcome drug resistance or reduce drug toxicity is of utmost importance. Indeed, it has been described that plant-derived compounds in combination with classical chemotherapeutic agents were more efficient in the treatment of TNBCs [112].

The most promising combination determined here was further explored, moving to what we called Phase 3, where another set of experiments was performed, including different types of assays for assessing cell viability (MTT and resazurin assays). Additionally, BrdU assay was included to evaluate the effects on cell proliferation. The new set of experiments was made in monolayer and, simultaneously, in multicellular aggregates (MCAs), which are 3D cell cultures. It is expectable that the other combinations that had no effect in monolayer would also have no effect in 3D culture, however, we cannot ensure this is the case because we did not test it.

In 3D culture models, cells are not attached to a plastic surface. Conversely, they form a three-dimensional cell arrangement. In the case of our study, the MCAs were self-assembled due to the use of low-attachment plates, and they were not grown in any matrix or scaffolds. These MCAs have cells in multilayers, which constitute a barrier to the penetration of chemicals to be tested [148], and therefore MCAs are considered a more realistic representation of an in vivo tumor for testing drug efficacy and toxicity [149]. For this reason, we selected higher Dox concentrations to be tested in the MCAs, to guarantee the drug effect.

The results obtained in this set of experiments (Phase 3) relative to monolayer cultures, replicated the results of Phase 2 in terms of cell viability, thus reinforcing the value of the obtained data. Both viability assays were concordant, because Fct alone did not present cytotoxicity while Dox (0.1 μM) plus Fct continued to cause effect, that significantly differed from the effects of both the compound and drug alone. Thus, Fct seemed to have potentiated Dox’s cytotoxicity. We consider these data as impressive in terms of future applications for cancer treatment, especially for TNBC: a low Dox concentration that alone did not have an effect on cell viability, when combined with a non-toxic compound, in this case, Fct significantly decreased cancer cells’ viability. This triad Fct/Dox/TNBC deserves further studies to explore the mechanisms involved in these interactions, namely using a pathway-focused gene expression analysis and additional cell-based assays. As to the latter, and because Fct may change the levels of reactive oxygen species (ROS) [87,141], the fine balance of which is critical for cancer cells to thrive, the determination of ROS would be a particularly relevant target in the future mechanistic assays.

Interestingly, the combination of Dox (0.1 μM) with Fct also had effects on cell proliferation, being more effective in inhibiting cell proliferation than the compounds in single exposure. Similar to what was seen in our study, Fct from two brown seaweeds induced antiproliferative effects on the MCF-7 cell line but using much higher concentrations than the ones applied here [150]. As we stressed earlier, factors such as the purity of freshly obtained isolates may as well promote inter-assay variability.

Despite the promising effects that we observed in monolayer cultures, they were not detected in 3D cultures. It was expectable that in 3D the same drug concentrations did not induce the same degree of cytotoxicity than in monolayer, as 3D cultures seem more resistant to drugs cytotoxicity [151,152], including Dox in 3D BC models [153,154]. Our results support this phenomenon. While in monolayer, Dox at 1 μM decreased cell viability by more than 50%, in 3D, a similar effect was only observed when the MCAs were exposed to Dox at 5 μM. The MCAs exposed to Dox from 0.1 to 2 μM did not show significant differences neither in viability nor in proliferation assays. As to the cell morphology, the qualitative stereomicroscopy and the measured areas corroborate the results of the bioassays. Only the MCAs exposed to Dox at 5 μM presented loose structures, correspondent to bigger areas. At this level, there were no differences between the drug alone and the combination with Fct, at any concentration.

However, when MCAs were observed after processing for light microscopy analysis and ICC, the drug effects started to be noticed at 1 μM, with a discrete but noticeable increase of cells with a morphology that was compatible with apoptosis; presenting cell shrinkage, nuclear condensation, chromatin margination, karyorrhexis, and putative apoptotic bodies [155,156]. These morphological aspects were confirmed by the increase of positive cells for caspase-3, indicating a higher number of apoptotic cells. With Dox at 2 μM, the effect was similar to Dox at 1 μM, but in the MCAs exposed to Dox at 5 μM the number of apoptotic cells greatly increased-over 80% of the cells stained positively for caspase-3. This high number of cells undergoing death caused a disaggregation of the MCAs’ structure and subsequent increase in their areas. There were no observed differences between the MCAs exposed to the drug alone and those subjected to its respective combination with Fct. As for cell proliferation, evaluated using Ki67, the control revealed a higher number of Ki67 positive cells, preferentially located in the outer part of the MCAs. In Dox and Dox combinations, there were still observed Ki67 positive cells, located in the inner part of the MCAs and not in the outer zone. There were no observed evident differences between Dox concentrations and even in Dox at 5 μM there were ki67 positive cells. In BrdU assay, differences in cell proliferation were only observed in Dox 5 μM and its combination with Fct.

From the structural evaluation in 3D culture, we conclude that the qualitative histology complemented with ICC, is an important and useful tool for evaluating the cytotoxicity of the tested drugs, as the effects were observable in more detail, revealing damages that were neither detected by the stereomicroscopic images, and respective measurements of areas, nor by the viability assays. Cell-based assays represent a technically simpler and quick way of assessing drug effects, while histological and ICC analyses are more time-consumable, require more equipment and know-how, and can be more expensive, especially due to the reagents for ICC; yet, they can give information on the localization of the processes through the MCAs. Furthermore, by using paraffin sections a great number of proteins related to different outputs, such as stem cell, epithelial-mesenchymal transition, and senescence markers can be detected. Our data calls for using histological analysis as an output to be included in drug testing.

## 5. Conclusions

This study tested the cytotoxic effects of five seaweed bioactive compounds (Asta, Fc, Fct, Lm, and Phg) alone and in a range of combinations with the reference drugs (Cis, Dox), in a panel of breast cell lines. We described concentrations in which the seaweed compounds alone presented cytotoxic effects against the BC cell lines. Also, conditions existed where a non-toxic concentration of the seaweed compound revealed potentiation or inhibition of the drug’s cytotoxicity. The results did not unveil patterns, varying according to the cell line, compound concentration used for the combination, and the drug in the combination. The overall findings showed that seaweed compounds may have anticancer effects against BC cell lines. However, studying and establishing an effective combinatory therapy is complex, with variability between cell lines and the used compounds and concentrations.

Among the tested compounds, Fct was the most promising compound concerning higher anticancer activity. Alone, Fct induced cytotoxicity at low concentrations against the three BC cell lines, without cytotoxic effects in the non-tumoral cell line. Also, in combination with Dox, it enhanced the drug’s effect under certain conditions. The data supported the importance of performing cytotoxicity screening in more complex culture models, as the effects found in monolayer were not reproducible in 3D, at least using the same bioassays. Our data stressed the importance of using other techniques, namely histological analysis, and ICC, for better understanding the cytotoxic effects and underlying mechanisms of seaweed bioactive compounds, alone and combined with drugs. Although there were no effects in the 3D model, the mixture of Dox with Fct, especially in TNBC needs further investigation, from increasing the concentration of Fct to recurring to other technologies for delivery of both types of chemicals.

## Figures and Tables

**Table 1:** Tested concentrations of bioactive seaweed compounds and reference drugs

Chemicals	Tested Concentrations
Astaxanthin	1; 10; 50; 100 and 200 µM
Fucoidan	10; 50; 100; 500 and 1000 µg/mL
Fucosterol	1; 2.5; 5; 7.5 and 10 µM
Laminarin	10; 50; 100; 500 and 1000 µg/mL
Phloroglucinol	10; 50; 100; 500 and 1000 µM
Cisplatin	0.1; 1, 10, 20 and 50 µM
Doxorubicin	0.001; 0.01; 0.1; 1 and 2 µM

**Table 2:** Concentrations of bioactive seaweed compounds and reference drugs used for combinations

	Drug	Dox (µM)			Cis (µM)		
Seaweed Compound		0.01	0.1	1	1	10	20
**Asta (µM)**	**10**	MCF7 SKBR3 MCF12A	MCF7 SKBR3 MDA-MB-231 MCF12A	MDA-MB-231	MCF12A	MCF7 SKBR3 MDA-MB-231 MCF12A	MCF7 SKBR3 MDA-MB-231
	**20**						
**Fc (µg/mL)**	**10**						
	**50**						
**Fct (µM)**	**1**						
	**5**						
**Lm (µg/mL)**	**10**						
	**50**						
**Phg (µM)**	**10**						
	**50**						

Asta: Astaxanthin; Fc: Fucoidan; Fct: Fucosterol; Lm: Laminarin; Phg: Phloroglucinol.

**Table 3:** Summary of the results on cell viability assessed by MTT of the combination seaweed bioactive compound and reference drugs after 72 h of exposure in monolayer

		Drug (µM)	Asta (µM)			Fc (µg/mL)			Fct (µM)			Lm (µg/mL)			Phg (µM)		
			0	10	20	0	10	50	0	1	5	0	10	50	0	10	50
**MCF7**	**Dox**	**0**															
		**0.01**															
		**0.1**															
	**Cis**	**0**															
		**10**															
		**20**															
**SKBR3**	**Dox**	**0**															
		**0.01**															
		**0.1**															
	**Cis**	**0**															
		**10**															
		**20**															
**MDA-MB-231**	**Dox**	**0**															
		**0.1**															
		**1**															
	**Cis**	**0**															
		**10**															
		**20**															
**MCF12A**	**Dox**	**0**															
		**0.01**															
		**0.1**															
	**Cis**	**0**															
		**1**															
		**10**															
	Control																
	Cell viability is significantly higher than the control																
	Cell viability is not significantly different from the control																
	Cell viability is significantly lower than the control																
	Cell viability is significantly different from control and from both drug and compound alone																

## Data Availability

The data are available on request from the corresponding author.
